# Dog Bites in Humans and Estimating Human Rabies Mortality in Rabies Endemic Areas of Bhutan

**DOI:** 10.1371/journal.pntd.0001391

**Published:** 2011-11-22

**Authors:** Tenzin, Navneet K. Dhand, Tashi Gyeltshen, Simon Firestone, Chhimi Zangmo, Chimi Dema, Rawang Gyeltshen, Michael P. Ward

**Affiliations:** 1 The Faculty of Veterinary Science, University of Sydney, Camden, Australia; 2 Regional Livestock Development Centre, Gelephu, Bhutan; 3 Phuentsholing General Hospital, Phuentsholing, Bhutan; 4 Jigme Dorji Wangchuk National Referral Hospital, Thimphu, Bhutan; 5 Gelephu Regional Referral Hospital, Gelephu, Bhutan; Swiss Tropical Institute, Switzerland

## Abstract

**Background:**

Dog bites in humans are a public health problem worldwide. The issues of increasing stray dog populations, rabies outbreaks, and the risk of dogs biting humans have been frequently reported by the media in Bhutan. This study aimed to estimate the bite incidence and identify the risk factors for dog bites in humans, and to estimate human deaths from rabies in rabies endemic south Bhutan.

**Methods:**

A hospital-based questionnaire survey was conducted during 2009–2010 among dog bites victims who visited three hospitals in Bhutan for anti-rabies vaccine injection. Decision tree modeling was used to estimate human deaths from rabies following dog bite injuries in two rabies endemic areas of south Bhutan.

**Results:**

Three hundred and twenty four dog bite victims were interviewed. The annual incidence of dog bites differed between the hospital catchment areas: 869.8 (95% CI: 722.8–1022.5), 293.8 (240–358.2) and 284.8 (251.2–323) per 100,000 people in Gelephu, Phuentsholing and Thimphu, respectively. Males (62%) were more at risk than females (P<0.001). Children aged 5–9 years were bitten more than other age groups. The majority of victims (71%) were bitten by stray dogs. No direct fatal injury was reported. In two hospital areas (Gelephu and Phuentsholing) in south Bhutan the annual incidence of death from rabies was 3.14 (95% CI: 1.57–6.29) per 100,000 population. The decision tree model predicted an equivalent annual incidence of 4.67 (95% CI: 2.53–7.53) deaths/100,000 population at risk. In the absence of post exposure prophylaxis, the model predicted 19.24 (95% CI: 13.69–25.14) deaths/year in these two areas.

**Conclusions:**

Increased educational awareness of people about the risk of dog bites and rabies is necessary, particularly for children in rabies endemic areas of Bhutan.

## Author Summary

Dog bites in humans are a public health problem worldwide. We conducted a hospital based questionnaire survey and described the incidence and risk factors for human dog bites in Bhutan. We also estimated the human death rate attributable to rabies in two rabies endemic areas of south Bhutan. Our study shows that dog bites incidents in humans are common in the survey areas. There were significant gender and age differences in bite incidents; males and the children are affected the most. The majority of the victims were bitten by stray dogs, increasing the risk of rabies infection if not treated in time. Our decision tree model predicted 2.23 (95% CI: 1.20–3.59) human deaths from rabies/year, equivalent to an annual incidence of 4.67 (95% CI: 2.53–7.53) deaths/100,000 in the two rabies endemic areas of south Bhutan. In the absence of post exposure prophylaxis, the model predicted 19.24 (95% CI: 13.69–25.14) deaths/year in these two areas. The public should be encouraged to visit hospitals for post exposure prophylaxis following dog bite injury in south Bhutan.

## Introduction

Dog bites in human are a serious public health problem and have been well documented worldwide [1], [2]. In the United States, 4.7 million people were estimated to have been bitten by dogs in 1994 (an incidence rate of 16.1/1000 in adults and 24.5/1000 in children), of whom 800,000 required medical treatment [3]. Later, a survey conducted during 2001–2003 in the USA estimated 4.5 million dog bites each year (an incidence rate of 16.6/1000 in adults and 13.1/1000 in children), an increase of 3% in adults and a decrease of 47% in children [4]. There have been similar reports of human dog bites in the United Kingdom [5], Belgium [6], Spain [7], Switzerland [8], Australia [9], India [10], and in the United Republic of Tanzania [11]. There are also several reports of dog bites incidents from other countries [12]–[17] but most cases are believed to be unreported, especially in developing countries.

The consequences of dog bites to humans are many. Although the most common issue is the direct physical injury, sometimes the injuries may cause permanent disfigurement of the victims requiring reconstructive surgery [4], [18], [19], psychological trauma and post traumatic stress [6], [20], [21], and rarely attacks can be fatal [2], [22]–[24]. Dog bites also result in a large monetary expense for treatment, emergency hospitalization and post-exposure treatment for rabies [1], [25]–[28]. For instance, the annual medical cost and other expenses associated with dog bites in the USA were estimated to be between $235.6 and $ 253.7 million in 1994 [28] while the French Postal Services reported 58,000 days of sick leave resulting from 3,357 bites to postal workers costing about US $ 2.5 million in 1985 [13]. Globally ≥15 million people receive rabies prophylaxis annually, mainly for dog bite injuries [29]. In addition, dog bite incidents also have direct impacts on the dogs involved in the bites, resulting in their relinquishment to shelters and euthanasia [21], [30]–[32]. Legislative action (e.g. Dangerous Dog Acts) have also been implemented in some developed countries to ban specific breeds of dogs because of the issue of increased bite incidents [33], [34]; such legislation does not appear to have been effective in reducing the incidence and severity of the bites. However, the severity of dog bite incidents is striking in developing countries: a vast majority of victims die from rabies infection. There are an estimated 55,000 human deaths annually, particularly in Asia and Africa, due to endemic canine rabies [35].

Like other countries, dog bites are common in Bhutan because of the presence of a large number of stray dogs in the streets [36], [37]. Although no cases of directly fatal dog attacks on humans have been documented, deaths due to rabies from dog bites have been reported in south Bhutan [38], [39]. Dog bites and the presence of rabies in the south border areas of Bhutan also results in substantial cost to the government [40], [41]. For example, approximately Bhutanese ngultrum (Nu.) one million was spent on rabies vaccine from 2002 to 2005 and the expenditure increased to about Nu.5.878 million in 2006 alone (Nu. 45 = 1 US$) [36], [42]. In addition, rabies outbreaks also cause a substantial cost to farmers from the deaths of farm animals as a result of spill over infection from dogs [40], [41]. Recently, there has been considerable media coverage on the stray dog population, the risk of dog bites to humans, and public nuisance in Bhutan [37], [42]–[46], yet there is no clear information about the epidemiology of human dog bites in Bhutan. Therefore, understanding the epidemiology of dog bites and the number of human deaths caused by rabies is important for public health planning program.

One of the approaches to understanding the scale of human deaths due to rabies is the use of decision tree models. This methodology has been developed by Cleaveland et al. [11] using active rabies surveillance data in Tanzania. The model is designed based on a series of probability steps using the distribution of bite injury on different body parts and the probability of developing rabies. More recently this decision tree model has been used by the World Health Organization to estimate human deaths from rabies in Asia and Africa overall. Globally, a total of 55,000 (90% CI: 24,000–93,000) human rabies deaths annually was predicted [35]. Fevre et al. [15] have also used this model to estimate human rabies deaths in Uganda using passive surveillance dog bite data. All these studies have provided clear information about the burden of rabies for proper planning program. In this study, we report the results of a dog bite survey conducted in three hospital areas of Bhutan. The objectives of this study were to:

1. estimate the incidence of human dog bites, describe characteristics of bites and to identify risk factors for dog bites in some areas of Bhutan;
2. understand the level of general knowledge and practice about rabies among bite victims; and
3. estimate the number of human deaths due to rabies in two hospitals areas of south Bhutan (rabies endemic areas) using a decision tree model and compare the estimates to the observed data.

The findings from this study are expected to guide dog bite and rabies prevention and control programs in Bhutan.

## Materials and Methods

### Study area and conduct of the survey

This study was conducted during 2009–2010 at three government medical hospitals in Bhutan – Jigme Dorji Wangchuk National Referral Hospital (JDWNRH), Gelephu Regional Referral Hospital (GRRH) and Phuentsholing General Hospital (PGH). These hospitals were selected because they provided the highest numbers of courses of rabies post exposure prophylaxis (PEP) to people during the preceding four years (2005–2008) [36]. JDWNRH is located in the capital city of Bhutan – Thimphu (interior western Bhutan), whereas GRRH and PGH are located in Bhutan–India border towns in the south-west and the south-central regions, respectively. The catchments of GRRH and PGH hospitals are endemic areas for rabies, whereas rabies has not been reported in dogs or other domestic animals in Thimphu (catchments area for JDWNRH) for at least 18 years [40]. Although rabies is not prevalent in the interior north of Bhutan, anti-rabies vaccination is normally administered to dog bite patients visiting hospitals for treatment due to the presence of rabies in south Bhutan [36].

In this survey, all dog bite victims who visited the injection section of these three hospitals to receive anti-rabies vaccine injections were interviewed using a pre-tested structured questionnaire designed to obtain information about the epidemiology of dog bite and bite-victim's knowledge about rabies. The questionnaire included closed questions about the demographics of the victims, circumstances of bite incidents, body parts injured and the degree of injury, ownership of biting animals, the level of knowledge about rabies, and post bite home treatment (washing of bite wound) prior to visiting the hospital for medical treatment. The interviews were conducted by the staff nurse of the respective hospitals providing PEP rabies vaccination, after the patients were prescribed rabies vaccine by the clinician on duty. The survey was conducted from 18 February 2009 to 8 February 2010 at PGH, 16 February to 20 September 2009 at JDWNRH, and from 11 February to 4 December 2009 at GRRH.

### Ethics statement

The purpose of the study was explained to each individual and they were informed that participation was voluntary and data collected were confidential. The participants who agreed to be interviewed were made to sign a consent form. The study was approved by the Ethics Committee of the Human Research and Epidemiology Unit, Ministry of Health, Bhutan (reference No. RESEARCH/PROPOSAL/08/8636).

### Data analysis

The questionnaire data were entered into a database (EpiInfo version 3.5.1). Descriptive analyses of the data were performed using a statistical software package (SPSS version 11.5, SPSS Inc, Chicago IL). Bhutan population census data from 2005 [47] were used to determine the population at-risk in the three hospital catchment areas. Dog bite incidence was calculated for each hospital catchment area and was expressed as the number of bite cases per 100,000 population at-risk. The initial plan to conduct the survey in each hospital for one year had to be modified due to logistical reasons. Therefore, because the survey period was variable between the three study areas (<12 months), the annual incidence for each hospital catchment area was estimated and was expressed as number of bite cases per 100,000 population at-risk per year.

The relationship between the number of bite cases and population density of the three hospital catchment areas according to age group was examined using the Spearman rank correlation test. Chi-square tests were used to compare the difference in proportions of dog bites between gender, age group and other variables. To make meaningful comparisons between age groups, observed frequencies of bites for various age groups were compared with expected frequencies calculated from the 2005 Bhutan census data [47]. For other variables, equal expected frequencies were assumed between groups. The variables of interest – such as occupation of the victims, ownership of dog involved in the bite incident, circumstances of bite incidents and knowledge about rabies – were compared among the three hospitals using Chi-square tests. A *p*-value of <0.05 was considered statistically significant.

In addition, standardized morbidity ratios (SMRs) were calculated for gender and age categories to examine whether there was any significant difference between the observed and the expected bite incidents in each category. This was expressed as: SMR = observed frequencies÷expected frequencies. The expected frequencies for each category were calculated using the 2005 catchment area census population for each hospital [47].

### Modeling human rabies deaths

We estimated human rabies deaths in the two hospital areas (Phuentsholing and Gelephu) of south Bhutan by using dog bite data and constructing a decision tree model developed by Cleaveland et al. [11]. Dog bite data from JDWNRH (interior west Bhutan, described in the descriptive analyses in this paper) was excluded from this model to avoid biased estimates since no rabies cases have been reported (either in dogs and humans) in that hospital region for at least 18 years.

**Figure 1:**
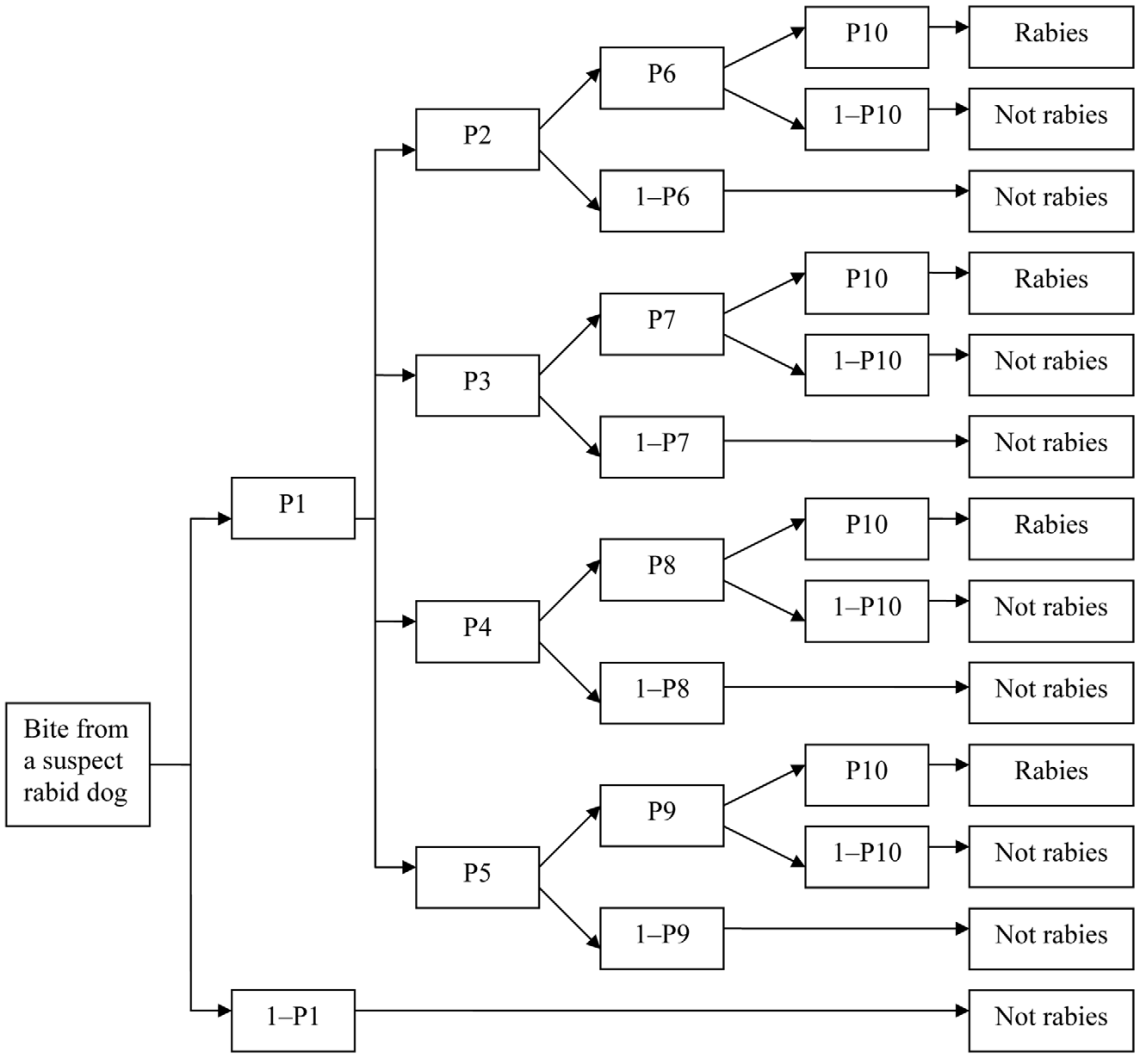
Decision tree model outlining the probability of rabies deaths. The model is adapted from Cleaveland et al., 2002 (reference number 11). Probabilities (P1–P10) are defined in Table 4 and described in the methods section. The probability calculated represents the probability of death following the bite of a suspect rabid dog.

**Figure 2:**
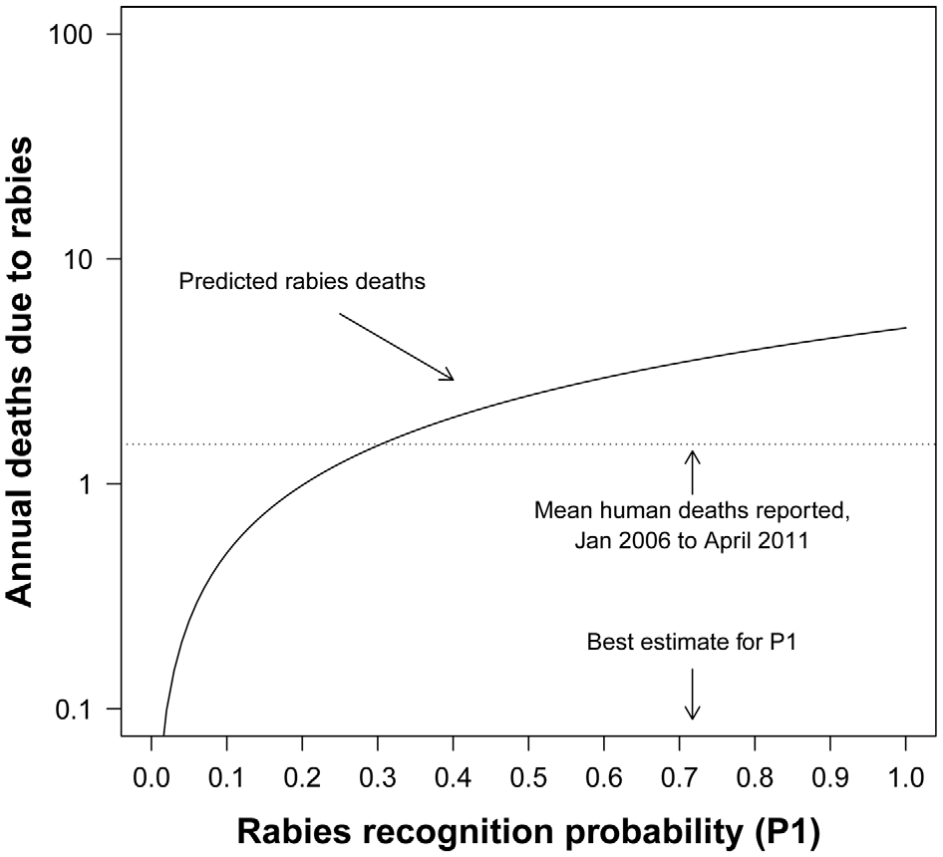
Predicted annual human deaths from rabies. Deaths are predicted in Phuentsholing and Gelephu areas of south Bhutan in relation to rabies recognition probability (*P1*) and mean number of human rabies deaths reported, adapted from Cleaveland et al., 2002 (reference number 11).

The decision tree model consists of 10 probability steps *(P1 to P10)* (Figure 1 and Table 1). The first step *P1* is the rabies recognition probability (the proportion of suspected rabid dog bites that are, in fact, rabid) [11]. In our survey dog bite victims had no knowledge about the status of the biting dog (whether rabid or not) and no biting dogs were traced back to observe their rabies status. Therefore, the disease status of the biting dogs was unknown. However, we used the proportion positive to rabies virus (by florescence antibody test) of rabies suspected dogs in these two areas for the period 1996 to May 2011. A total of 46 dog brain samples were collected from these two hospital areas and submitted to the Veterinary Laboratories in Bhutan for rabies virus confirmation. Of these, 33 (72%) samples were positive for rabies virus by FAT. Therefore, the rabies recognition probability (*P1*) in dogs was estimated to be 72% for this analysis (Table 1). In addition, a sensitivity analysis was conducted on *P1* using different rabies recognition probabilities to explore the impact on the final model output (Figure 2). The probability of rabies recognition was 68% (17/25) in Tanzania [11], 51% (43/85) in Kenya [48], 42% to 77% in Uganda [15], and between 38% to 50% in Asia and 64% in Africa [35].

**Table 1:** Model parameters, probability distributions and data sources used in the prediction of human deaths from rabies in Phuentsholing and Gelephu areas of south Bhutan from dog bite survey data.

Parameter	Description	Probability and distribution	Data source
P1	Probability of a suspected rabid dog being confirmed rabid on laboratory diagnosis (33/46)	Binomial: p = 0.720; n = 46	Field data, [49]
P2	Bite injury to the head or neck	Point estimate: (11/193) = 0.057	Field data
P3	Bite injury to the hand or arm	Point estimate: (41/193) = 0.212	Field data
P4	Bite injury to the trunk	Point estimate: (3/193) = 0.016	Field data
P5	Bite injury to the leg or foot	Point estimate: (137/193) = 0.715	Field data
P6	Probability of developing rabies following a bite injury to the head by a rabid dog	Triangular: minimum = 0.30, mode = 0.45, maximum = 0.60	[11],
P7	Probability of developing rabies following a bite injury to the hand or arm by a rabid dog	Triangular: minimum = 0.15, mode = 0.28, maximum = 0.40	[11], [15], [35]
P8	Probability of developing rabies following a bite injury to the trunk by a rabid dog	Triangular: minimum = 0.00, mode = 0.05, maximum = 0.10	[11], [15], [35]
P9	Probability of developing rabies following a bite injury to the leg or foot by a rabid dog	Triangular: minimum = 0.00, mode = 0.05, maximum = 0.10	[11], [15], [35]
P10	Probability of an individual receiving post exposure treatment if bitten by a suspected rabid dog (see methods)	Triangular: minimum = 0.80, mode = 0.90, maximum = 0.95	[36]

For the *P2*–*P5* probability steps, the dog bite injury data were classified according to the distribution of bites on different body parts: head/neck (*P2*); hand/arms (*P3*), trunk (*P4*), and legs/thigh (*P5*) (Figure 1 and Table 1); and the age group of the victims: 0–4; 5–9; 10–14, and >15 years of age (Table 2). The point estimates (proportion of bites) on each body part and according to each age group were then calculated using the dog bite data (Tables 1 and 2). The probability (*P6*–*P9*) of developing rabies following the bite of a rabid dog to the head (*P6*), arms (*P7*), trunk (*P8*) and legs (*P9*) were 45%, 28%, 5% and 5%, respectively [11], [15], [35] (Table 1).

**Table 2:** Distribution of bite injuries on the body according to age group of dog bite patients in Phuentsholing and Gelephu hospital areas of south Bhutan.

Age group (years)	Head/neck	Hand/arms	Trunk	Legs/thigh	Total
0–4	6	3	1	13	23
Point estimate	0.261	0.130	0.043	0.565	1
5–9	2	11	1	35	49
Point estimate	0.041	0.224	0.020	0.714	1
10–14	1	3	1	28	33
Point estimate	0.030	0.091	0.030	0.848	1
>15	2	24	0	62	88
Point estimate	0.023	0.273	0.000	0.705	1

In the case of multiple bites, the site of the most severe bite is given. The row proportions (point estimate) are calculated for each group.

The last step, the probability of receiving post exposure treatment (*P10*) was determined on the basis of previously published data [36]. There is a very high probability of people receiving PEP in these areas of Bhutan due to endemicity of rabies, rabies vaccine being freely available in the hospitals, easy accessibility of these hospitals (centrally located in the towns) and due to free medical services. Our previous study [36] on the use of PEP in Bhutan showed that large doses of anti-rabies vaccination were freely provided to all patients with dog bite injuries (even in the interior of Bhutan where rabies is not present) and also to all the WHO categories of exposure (I, II and III) [29]. To account for this in the model, we used the following as the probability of receiving PEP following a bite from a suspected rabid dog in these two study areas: minimum = 80%, most likely = 90% and maximum = 95% (Table 1).

The probability of dying of rabies following a bite from a suspected rabid dog was then calculated from the probability parameters using the formula [11] *Pdeath (probability of death) = P1×((P2×P6)+(P3×P7)+(P4×P8)+(P5×P9))×(1−P10).* Then the total number of deaths (Tdeath) caused by rabies per year in these two hospital region were calculated using the formula [11] *Tdeath = (I×Q×Pdeath/100 000)*, in which ‘*I*’ is the incidence of suspected rabid dog bites per 100,000 population at risk per year and ‘*Q*’ is the total population at-risk (n = 47721) which was based on the 2005 population and housing census data of Bhutan [47]. The confidence limits for the total number of deaths from rabies were calculated by assigning the probability distribution to the inputs parameters [11] and running Monte Carlo simulations for 10,000 iterations using R software (version 2.12.0 (210-10-15), R Development Core Team, http://www.r-project.org). The mean and the 95% confidence interval were estimated. The total number of human deaths due to rabies was also estimated in each age category of dog bite victims on the basis of the bite injury distribution on the body (Table 3).

**Table 3:** Annual predicted death counts and incidence rate from rabies for different age groups in Phuentsholing and Gelephu areas of south Bhutan, calculated using the decision tree model.

Age group	Annual predicted death counts from rabies in humans (95% Confidence interval)	Predicted deaths from rabies in humans/100,000/year (95% Confidence interval)
0–4 years	0.35 (0.20–0.55)	7.43 (4.15–11.62)
5–9 years	0.48 (0.26–0.77)	9.16 (4.90–14.72)
10–14 years	0.23 (0.11–0.38)	4.58 (2.25–7.70)
>15 years	0.88 (0.47–1.42)	2.69 (1.43–4.34)

## Results

A total of 339 patients were interviewed at the three hospitals. Fifteen questionnaires were excluded from the analyses because two were incomplete and 13 were related to cat (n = 8) and other animal bite (n = 5) injuries. The final analysis was undertaken on 324 dog bite questionnaires, but not all questions were completed and so the sample size differed for each question analyzed.

### Bite incidence

A total of 131, 100 and 93 dog bite victims were reported to JDWNRH, GRRH and PGH, respectively, for post bite rabies vaccination during 2009–2010. The incidence of bites differed significantly (P<0.001) between the hospital areas: 869.8 (95% CI, 722.8–1022.5), 293.8 (240.9–358.2) and 284.8 (251.2–323.0) per 100,000 population per year in Gelephu, Phuentsholing and Thimphu, respectively.

### Age and gender of the victims

**Figure 3:**
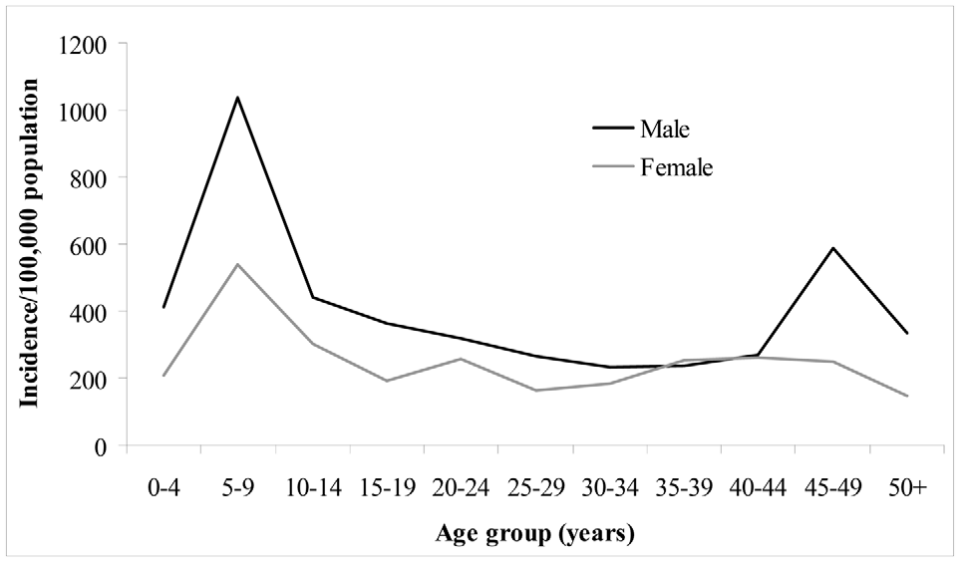
Annual incidence of dog bites/100,000 population classified by age and gender. Data is based on a survey of dog bite victims attending three hospitals (Jigme Dorji Wangchuk National Referral hospital, Phuentsholing General hospital, Gelephu Regional Referral hospital) in Bhutan, 2009–2010.

There were significantly more bite cases in males (201/324, 62%) than females (123/324, 38%) (χ^2^ = 18.78; P<0.001). Males were 1.15 times (95% CI 1.00–1.32) more likely to report dog bites than expected, compared to females (P = 0.053) (Table 4). The median age of all bite cases was 17.5 years and the modal age was 6 years (mean 21.2 years; range: <1 to 80 years). There were significant differences in the proportion of bite victims between various age categories (χ^2^ = 73; P<0.001). Approximately two-thirds of the bite cases were reported in people <25 years of age. However, those in the age groups 5–9 years were the most common victims of dog bites (74/324; 23%) and were 2.3 times (95% CI 1.83–2.88) more likely to report dog bites than expected, compared to adults or other age categories (P<0.001) (Table 4). There was also significant correlation between dog bite incidents and population density in the three hospital catchment areas according to age group (r_s_ = 0.77; P = 0.005). Figure 3 illustrates the age and gender distribution of dog bites and shows that the incidence of dog bites was greater amongst children aged 5–9 years and greater in males than females across all age groups.

**Table 4:** Standardized morbidity ratio (SMR) of reported dog bite incidents according to gender and age of victims in three hospital catchment areas (Jigme Dorji Wangchuk National Referral hospital, Phuentsholing General hospital, Gelephu Regional Referral hospital) in Bhutan, 2009–2010.

Variables/categories	N	Percent	SMR*	95% Confidence interval	*P*-value
Gender					
Female	123	38	0.83	0.69–0.98	0.030
Male	201	62	1.15	1.00–1.31	0.053
Age group (years)					
0–4	31	10	0.97	0.67–1.35	0.882
5–9	74	23	2.31	1.83–2.88	<0.001
10–14	40	12	1.14	0.83–1.54	0.393
15–19	27	8	0.73	0.50–1.04	0.089
>20	152	47	0.80	0.68–0.94	0.005

N = number of reported dog bite victims in each group.

*SMR >1 means that they are more likely to report bites than expected, a value <1 means that they are less likely to report bites than expected and a value of 1 means that they are equally likely to reported than expected. Children in age group 5–9 are 2.3 times more likely to report dog bites than expected comparing to other age groups.

### Occupation of the victims

A significant difference (χ^2^ = 138.44; P<0.001) in the proportion of bites was observed between the various occupational groups of the victims. School children were the most common (45%) victims of dog bites. There were also significant differences among the three hospital catchment areas with respect to the number of bite cases among the various occupational groups (χ^2^ = 39.83; P<0.001), with school children reporting more bites than other occupational groups in JDWNRH, PGH and GRRH (Table 5).

**Table 5:** Comparison of occupation and other responses to a questionnaire of dog bite victims attending three hospitals (Jigme Dorji Wangchuk National Referral hospital, Phuentsholing General hospital, Gelephu Regional Referral hospital) in Bhutan, 2009–2010.

	Hospital				
Variables/categories	JDWNRH (N, %)	PGH (N, %)	GRRH (N, %)	Total (%)	*P*-value
Occupation					<0.001
Housewives/businessman	19 (14)	13 (14)	2 (2)	34 (10)	
Employees	42 (32)	16 (17)	9 (9)	67 (21)	
Farmers	11 (8)	7 (7)	16 (16)	34 (10)	
Preschool children	12 (9)	17 (18)	14 (14)	43 (13)	
School children	47 (36)	40 (43)	59 (59)	146 (45)	
Ownership of dogs involved in the bites					0.047
Own dogs	40 (31)	18 (19)	35 (35)	93 (29)	
Stray dogs	91 (69)	75 (81)	65 (65)	231(71)	
Circumstances of bites (was the bite provoked?)					0.183
Yes	26 (20)	19 (23)	10 (12)	55 (19)	
No	104 (80)	64 (77)	72 (88)	240 (81)	
Availability of biting dog for observation					0.052
Yes	45 (35)	19 (21)	35 (36)	99 (31)	
No	85 (65)	71 (79)	63 (64)	219 (69)	

### Ownership of dogs involved in the bites

The victims were predominantly bitten by stray dogs (231/324; 71%), rather than by owned dogs (93/324; 29%) (χ^2^ = 58.77; P<0.001). Of the owned dogs, 71 cases were bitten by a neighbor's dog. A significant difference among the three hospital catchment areas was observed with respect to the bite incidents by owned and stray dogs (χ^2^ = 6.124; P = 0.047), with the largest difference (81 versus 19%) occurring in PGH (Table 5).

### Circumstances of the bite incident

Most bites were reported to be unprovoked (240/295, 81%) rather than provoked (55/295, 19%) (χ^2^ = 116.02; P<0.001). However, there were no significant differences between the circumstances of bites incidents reported in the three hospitals (χ^2^ = 3.39; P = 0.183) (Table 5).

### Anatomical site of bite and injury type

**Figure 4:**
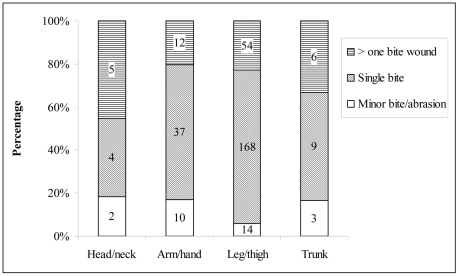
Anatomic location and severity of dog bite wounds. Data is derived from victims attending three hospitals (Jigme Dorji Wangchuk National Referral hospital, Phuentsholing General hospital, Gelephu Regional Referral hospital) in Bhutan, 2009–2010 (the number in the figure indicates the number of bite victims).

**Figure 5:**
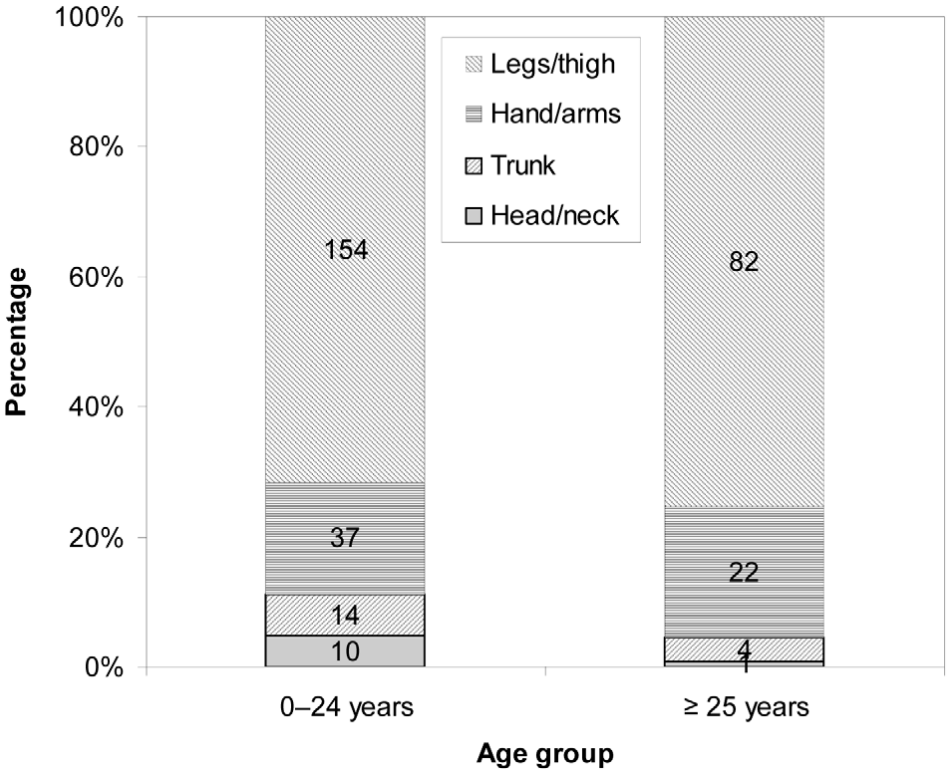
Anatomic location of dog bite wounds according to age group. Data is derived from victims attending three hospitals (Jigme Dorji Wangchuk National Referral hospital, Phuentsholing General hospital, Gelephu Regional Referral hospital) in Bhutan, 2009–2010 (the number in the figure indicates the number of bite victims). Age was categorized into the two groups indicated.

Most (90%) dog bites were inflicted on the extremities with 73% on the legs and 18% on the hand/arms. There was a significant difference between the bite incidents and the anatomic sites (χ^2^ = 412.07; P<0.001). However, a majority of the bites were single bite injuries (218/324, 67%). There was also a significant difference between the severity and anatomic locations of bite wounds (χ^2^ = 15.18; P<0.019) (Figure 4). Figure 5 illustrates the anatomic location of dog bite wounds according to the age group of the victim. The lower extremities (leg/thigh) were the most common site of bite in all age groups and no significant difference was observed between these two age groups (0–24 versus ≥25 years) with respect to different anatomic bite sites (χ^2^ = 4.05; P = 0.212). However, no case of a fatal dog bite injury was reported during the study period.

### Status of dogs involved in the bite and availability for observation

Of the 320 respondents, a majority (189, 59%) of the victims mentioned that the disease (rabies) status of the dogs involved in the bite incidents was unknown, 101 victims (32%) mentioned that the biting dog looked normal, and 30 victims (9%) mentioned that the biting dogs were suspected of rabies. However, a majority of the victims responded that the biting dog was not available for observation (219/318, 69%). Three respondents mentioned that the dog involved in the bite was killed. There was only borderline significant differences among the three hospital catchments areas with respect to the response of the victims that the dog was unavailable for observation (χ^2^ = 5.911; P = 0.052).

### Seasonal distribution of bites

Dog bite incidents were reported throughout the year with more bite incidents during the spring months (March–May) (129/324; 40%) followed by winter months (December–February) (90/324; 28%) and autumn (September–November) (72/324; 22%). The reported incidents were lowest during the summer months (June–August) (33/324; 10%). There were significant differences in the proportions of bite incidents between the seasons (χ^2^ = 58.88; P<0.001).

### Previous history of dog bite

A previous history of dog bite was reported by 40 victims: 33 persons were bitten twice, five persons were bitten three times, and two persons were bitten four times.

### Knowledge about rabies

Of the 318 respondents, a majority (263, 83%) of the victims or the guardian/parents of the victims (in minor cases) was aware of and had heard about the fatality of rabies. There was a significant relationship between the hospital catchment areas with respect to knowledge about rabies (χ^2^ = 34.26; P<0.001). The proportion of bite victims from the rabies endemic areas reported to two hospitals (GRRH and PGH) were more aware of rabies compared to victims reported to JDWNRH. A majority (277/305, 91%) of the victims were also aware that rabies can be prevented and controlled by regular vaccination of dogs (χ^2^ = 203.28; P<0.001). However, no significant difference was observed among the three hospitals with respect to the dog bite victims' knowledge that rabies can be prevented by vaccination of dogs (χ^2^ = 1.054; P = 0.590) (Table 6).

**Table 6:** Comparison of knowledge and practices about rabies prevention based on the response of dog bite victims attending three hospital areas (Jigme Dorji Wangchuk National Referral hospital, Phuentsholing General hospital, Gelephu Regional Referral hospital) in Bhutan, 2009–2010.

	Hospital catchment areas				
Variables/categories	JDWNRH (N, %)	PGH (N, %)	GRRH (N, %)	Total (%)	*P*- value
Had heard of rabies					<0.001
Yes	89 (69)	78 (91)	96 (96)	263 (83)	
No	40 (31)	8 (9)	4 (4)	52 (17)	
Believe that regular vaccination of dogs can prevent rabies					<0.001
Yes	116 (91)	69 (88)	92 (93)	277 (91)	
No	12 (9)	9 (12)	7 (7)	28 (9)	
Had washed dog bite wound with soap and water					<0.001
Yes	72 (59)	27 (29)	73 (74)	172 (55)	
No	50 (41)	65 (71)	26 (26)	141 (45)	

### Bite wound washing at home

Of the 313 respondents, 141 victims (45%) had washed their bite wound with soap and water at home before presenting to the hospital. However, there were no significant differences in the proportions of those who washed and those that did not wash the bite wound at home (χ^2^ = 3.07; P = 0.08) (Table 6).

### Rabies post exposure prophylaxis

All dog bite victims were given rabies post exposure prophylaxis (vaccine) on the first day (day 0) of their visit to the hospital, irrespective of the epidemiological likelihood of the implicated dog being rabid and the local epidemiology of rabies. The patients were believed to have been advised to complete the course (5 doses) since Bhutan follows the 5-dose intramuscular regimen (36) [29]. However, no post exposure vaccine course data (subsequent vaccine series dada) were collected for our analysis since our earlier study on PEP vaccine use had provided important information about the epidemiologic characteristics of rabies post exposure prophylaxis in Bhutan [36].

### The prediction of human rabies death

From 2006 to April 2011, there were eight reported human deaths due to rabies in Gelephu (n = 4) and Phuentsholing (n = 4) hospital areas, which accounted for a cumulative incidence of 16.76 deaths/100,000 population. The mean annual number of reported human deaths due to rabies (from 2006 to April 2011) in these two areas were 1.5 (95% 0.75–3.00), equivalent to an annual incidence of 3.14 (95% CI: 1.57–6.29) per 100,000 population. Based on our dog bite survey data, the model predicted 2.23 (95% CI: 1.20–3.59) deaths per year, equivalent to an annual incidence of 4.67 (95% CI: 2.53–7.53) deaths/100,000 population in Gelephu and Phuensholing areas of south Bhutan. Table 3 summarizes the predicted death distribution by age group in these two hospital catchment areas (Gelephu and Phuesholing), and shows that annual human predicted deaths from rabies per 100,000 population were greater for ages <15 years. In the absence of any post exposure treatment, the 223 bite incidents would result in a total of 19.24 (95% CI: 13.69–25.14) deaths per year in these two areas of Bhutan, equivalent to an annual incidence of 40.31 (95% CI: 28.70–52.68)/100,000 populations. Figure 3 shows the predicted annual human deaths due to rabies in the two hospital areas (Gelephu and Phuentsholing) of south Bhutan in relation to different rabies recognition probabilities (*P1*) and the mean number of deaths reported between 2006 and April 2011.

## Discussion

The annual incidence of dog bites was higher in the GRRH catchment area than in the other two hospital catchment areas. This difference could be explained by the geographical location of the individual study areas, population density and the local epidemiology of rabies. It is important to note that the GRRH catchment area is located in the south-central area of Bhutan, which includes towns adjacent to Indian towns across the international border, and has experienced frequent rabies outbreaks [49]. It is likely that the dog bite victims (irrespective of the disease status of biting dogs – whether rabies suspect or normal healthy dogs or pet dogs) might have reported to the GRRH for medical treatment because of a fear of rabies. In addition, high dog population density and trans-border movement of dogs (particularly stray dogs in such border towns) could be another reason for the high incidence of dog bites. A recent media report indicates that about 500 people visited GRRH for dog bite injuries treatment during 2010 [50] which greatly exceeds the number we recorded in our study. Reported dog bite incidents have been increasing, as have human deaths from rabies infection [38], [51].

The risk factors for human dog bites identified in this study are very similar to those of other studies conducted elsewhere, mostly in developed countries [1]. For instance, dog bite injuries were more common in children, particularly those aged 5–9 years, and more common in males than females. A previous study on post exposure rabies events in humans in Bhutan also showed that PEP were provided more often to younger age groups and to males [36]. In general, our results are in agreement with those from several studies conducted both in developed [1], [3], [7], [25], [27], [52]–[54] and developing [10], [16], [17], [26], [55], [56] countries. Increased dog bite incidents in children is considered a behavioral risk because of their extreme curiosity, lack of inhibition, limited knowledge and experience about dog behavior, and inability to protect themselves from an attack [1], [3], [27], [52], [57]. It has also been suggested that bites in children are more likely to be reported than in adults because of more parental concern towards children or the severity of their injuries [3]. However, it is also believed that children in developing countries do not report minor bites or scratches to their parents, which increases the risk of rabies infection [58].

Animal bites usually occur as a result of provocation by the victims during play and by abusing/teasing the animal, repeated irritation or as unprovoked bites in which people are attacked [1], [59]. Our results show that a majority of the dog bites occurred as an unprovoked bite (76%) in which people were attacked and, mostly by stray dogs (68%). This suggests that the presence of a high density of dogs on the street (commonly seen in developing countries, including Bhutan) [55], [56], [60] is a risk factor for increased reports of dog bites incidents in Bhutan. It has been suggested that human behaviors not generally regarded as provocative can frighten dogs or may be misinterpreted by some dogs as an invasion of their territory and may incite an attack [1]. It is also important to note that the high number of unprovoked bites in this study may be due to biased opinions given by the victims. However, rabid dogs (in rabies endemic countries) would be aggressive and bite people indiscriminately without any provocation.

Dog bite injuries to the lower extremities were more common (72%) than to other body parts, irrespective of the age of the victim in this study. This result is in contrast to some other studies in which more bite injuries were reported to the head, neck and face [1], [3], [7], [21], [25], [27], [61], [62]. However, this difference may be explained by the ownership of the biting dogs, the physical environment of the bite incidents and the study areas. In the developed world, pet dogs (owned dogs or neighbors' dogs) – which are known to the victim – are most commonly involved in bite incidents to the head, neck and face. This may be due to the short stature of children and playful interaction with pets – kissing, hugging and petting [1], [3], [7], [21], [61], [63]. Our study showed that people were more commonly bitten by stray dogs and bites occurred more commonly to the lower extremities. Similarly, some other dog bite studies in developing countries have shown that stray dogs were commonly involved in bites to the extremities [10], [55], [56], which is in agreement with our findings. It is also likely that the victims (e.g. children) would have used a hand or leg to abuse/tease the dogs or to separate fighting dogs or defending dog attacks, resulting in more bites on the extremities [7], [64]. Rabid dog bites to the upper body and extremities (head, neck, arm, hand) are more dangerous than bites to the lower extremities. The median risk of death following rabid dog bites to the head, hand, trunk and legs have been reported to be 45%, 28%, 5% and 5%, respectively [11], [15], [35].

Our study showed that dog bite incidents occurred throughout the year, with increased cases from late winter to mid-spring (February through April). It is difficult to correlate factors that might explain this peak during this period in Bhutan. However, there is a possible bias in estimated annual dog bite incidence in this study because the survey could not be conducted for one full year within two of the survey areas due to logistical constraints, and dogs bites might have seasonal variability. Studies in developed countries have reported that most dog bite incidents occur during the spring and summer months [3], [6], [21], [27], [65]. Such observations have been explained by behavioral changes: more interaction between pets and children during the warmer months with less parental supervision, thus increasing the risk of bite incidents [6]. In Thailand, reports of dog bite incidents in children increased during the months of March–May and October, the period of school vacation [26].

Understanding people's level of knowledge about dog bites and the risk of potential zoonotic disease transmission – particularly rabies – is important for planning an awareness education program. In this study, the majority (81%) of dog bite victims (or the parents/guardian of minors) were aware of rabies, which is in agreement with the results from some other studies in Asia [66]–[69]. However, the respondents that reported to two hospitals (GRRH and PGH) in south Bhutan were more aware of rabies than respondents who reported to JDWNRH in Thimphu. This difference is expected because the south is an endemic region for rabies with frequent reports of outbreaks; the people might have previously seen rabies cases in dogs and farm animals, or might have heard about rabies from family, friends or the news media [49]. The interior of Bhutan is free of rabies and people may not be aware of the disease. On the contrary, most victims (52%) reportedly did not wash their wound with soap and water at home before visiting the hospital for medical treatment. This finding suggests that a proper health educational program on rabies and wound care at home [29], [70] is required. Cleaning and flushing of the bite wound with soap and water immediately after being bitten is one of the most important steps recommended by the WHO. This procedure will remove much of the rabies virus from the wound and may considerably reduce the risk of contacting rabies (if the biting dog is infected with rabies) [29], [71].

Predicted human deaths due to rabies from the decision tree model were almost the same as the annual mean human rabies deaths reported in these two study areas, indicating that there is no serious under-reporting of rabies in Bhutan. The fatal nature of the disease (with classic rabies symptoms), availability of free medical services and accessibility to the hospitals might be the main reasons for good reporting of human rabies deaths in Bhutan, but some extent of under reporting of dog bites may be possible. The model also predicted that in the absence of any post-exposure treatment, the annual dog bite counts of 223 would result in a total of 19.24 (95% CI: 13.69–25.14) deaths per year in these two areas of Bhutan, which is equivalent to an annual incidence of 40.31 (95% CI: 28.70–52.68) deaths per 100,000 population. Therefore, human rabies PEP is important for rabies prevention in Bhutan. On the basis of laboratory examination of submitted samples in these two areas, a rabies recognition rate of 72% was used in this study. However, the proportion of the victims bitten by a confirmed rabid dog is largely unknown since tracing of the source of biting dogs and confirmation of rabies is not usually done. Active surveillance of bite injuries and tracing of the biting dogs would provide clear information about the public health hazard of rabies. Nevertheless, with high recognition probability of rabies in dogs in these areas, it is important to make people aware of the danger of rabies and encourage reporting to hospitals for post bite treatment. It is important to note that we have estimated human deaths from rabies in two areas of south Bhutan that are endemic for rabies. Accordingly, the human population at-risk for canine rabies was also assumed to be the number of people living within these two hospital catchments areas. We did not include the entire population of Bhutan in order to avoid bias estimates, since rabies cases have not been reported in the interior of Bhutan.

In conclusion, this study has provided important information about human dog bites, risk factors and the burden of rabies in Bhutan. The presence of large numbers of stray dogs is a public health issue in Bhutan. Intervention measures should include public educational programs on dog behavior, dog-child interaction, and the importance of responsible dog ownership, particularly in children [72], [73]. Lessons on dog behavior, the risk of dog bites, bite wound management (e.g. washing with soap and water) and rabies can also be integrated into the elementary school curriculum to educate children on the public health hazard of dog bites [58]. In a randomized control trial of an educational intervention for the prevention of dog bites in children in Australia, Chapman et al. [73] demonstrated that children who had been educated and provided information on ways to approach dogs displayed appreciably greater precautionary behaviors than children that did not receive any awareness education on dog behaviors and intervention. Therefore, dog bite preventive education is important in children. Similarly, enforcement of regulations for licensing of dogs and rabies vaccination, stray dog population management and animal birth control programs are important to reduce the bite incidents and post bite treatment cost [72], [74]. One study in Spain has shown a significant decline in hospitalizations caused by dog bites after enactment of stricter regulations on dog ownership [74]. This suggests that a regulatory approach may also help in reducing dog bite injuries in addition to other educational programs. Continuing surveillance of dog bites is necessary to detect trends and evaluate the effect of prevention efforts. For this, a national dog bite database and reporting system implemented through local primary health care centers may be appropriate for the surveillance and monitoring of dog bite incidents in Bhutan.
